# Historical significance and taxonomic status of *Ischyrodon meriani* (Pliosauridae) from the Middle Jurassic of Switzerland

**DOI:** 10.7717/peerj.13244

**Published:** 2022-04-07

**Authors:** Daniel Madzia, Sven Sachs, Christian Klug

**Affiliations:** 1 Department of Evolutionary Paleobiology, Institute of Paleobiology, Polish Academy of Sciences, Warsaw, Poland; 2 Abteilung Geowissenschaften, Naturkunde-Museum Bielefeld, Bielefeld, Germany; 3 Paläontologisches Institut und Museum, Universität Zürich, Zürich, Switzerland

**Keywords:** Thalassophonea, Pliosauridae, Plesiosauria, Callovian, Middle Jurassic, Switzerland

## Abstract

*Ischyrodon meriani* is an obscure pliosaurid taxon established upon an exceptionally large tooth crown of a probable Callovian (Middle Jurassic) age that originates from Wölflinswil, Canton of Aargau, Switzerland. Despite being known for almost two centuries, the specimen remains poorly researched. Historically, *I. meriani* has been associated, or even considered conspecific, with *Pliosaurus macromerus* and *Liopleurodon ferox*. However, neither of the two hypotheses have been tested through detailed comparisons or using modern quantitative methods. Here, we redescribe the type of *Ischyrodon meriani*, illustrate it, and compare to teeth of thalassophonean pliosaurids, with special focus on Jurassic representatives of the clade. Multivariate analyses show close similarities to *L. ferox* but comparable structures to those of *I. meriani*, including a distinctive pattern of the apicobasal ridges, are also observable in some mid-Cretaceous brachauchenines from the ‘*Polyptychodon*’ assemblage of East and South East England. While it is likely that *I. meriani* represents a *Liopleurodon*-like taxon, or is indeed conspecific with *L. ferox*, which would make *I. meriani* the proper name for the species, any such taxonomic considerations are hindered by the fragmentary nature of the type specimens of both these taxa as well as limited knowledge of the dental variability within and between individual jaws of *L. ferox*. Currently, *I. meriani* is best treated as a *nomen dubium*. Finally, we discuss the potential implications of *I. meriani* being conspecific with *L. ferox*, and additionally provide a commentary on the taxonomic status of *Liopleurodon*.

## Introduction

*Ischyrodon meriani* is a name pertaining to an obscure pliosaurid taxon, first mentioned in Hermann von Meyer’s article titled “Mittheilungen, an Prof. Bronn gerichtet” (“Contributions to Prof. Bronn”) and published on July 26th, 1838, in *Neues Jahrbuch für Mineralogie, Geognosie, Geologie und Petrefaktenkunde* (von Meyer, 1838). The journal was edited at that time by K.C. von Leonhard and H.G. von Bronn and the contributions that were being published there basically represented letters informing the editors about newly discovered fossil specimens, which were often introduced as new taxa. However, neither in-depth descriptions nor illustrations were usually provided.

*Ischyrodon meriani* was described based upon an exceptionally large (∼110 mm high) tooth crown (NMB L.D.37) originating from the Middle Jurassic of ‘Wölffiswyl’ (Wölflinswil in modern spelling) in Canton of Aargau, Switzerland. Von Meyer learned about the specimen, along with other fossil materials, from Peter Merian, professor of physics, chemistry, geology, and paleontology at the University of Basel (Switzerland). Merian considered the specimen to be a new taxon and also coined the name *Ischyrodon*, though he has never published it himself. Eventually, von Meyer (1838) introduced the name *Ischyrodon Meriani* [sic] for the specimen. Still, despite being mentioned in several papers and text books in subsequent years (von Meyer, 1841; Hoffmann, 1845; Geinitz, 1846; Giebel, 1847; Pictet, 1853), the tooth crown had not been described and illustrated until 18 years after the publication of the initial article in which the taxon was named (von Meyer, 1856a).

The taxonomy of *I. meriani* has always been challenging. Von Meyer (1841) noticed that the *Ischyrodon* tooth resembles the teeth of *Thaumatosaurus* from the Middle Jurassic of Neuffen (Germany). Similarities between the tooth crown of *I. meriani* and the teeth of *Thaumatosaurus* and *Polyptychodon* were further discussed in the full description of *I. meriani* by von Meyer (1856a). At that time, however, it still remained unclear as to what group these animals belonged to. Kiprijanow (1883) considered *Ischyrodon* to be closely related to *Pliosaurus*, and included it, together with *Polyptychodon* and *Thaumatosaurus*, within a new group of Sauropterygia which Kiprijanow (1883) called Thaumatosauria. Von Quenstedt (1885) listed *Ischyrodon meriani* among the Plesiosauri [sic] and highlighted similarities between the tooth crown and the teeth of *Pliosaurus* and *Polyptychodon*. Zittel (1887–1890) considered it similar to *Pliosaurus*, whereas Lydekker (1889) formally listed it as a synonym of *Pliosaurus*, noting it possibly represents *P. macromerus*. Lydekker (1889: 132) wrongly regarded *Ischyrodon* to be from the Upper Jurassic of Würtemberg [sic], in southern Germany, and stated that it “presents all the characters of the teeth of the English Kimeridgian Pliosaurs”, and added that “from its large size [it] may be provisionally referred to [*Pliosaurus macromerus*]”. Lydekker (1889: 132) concluded that, “[i]f this reference [is] correct the name *P. meriani* should supersede *P. macromerus*”. Following Lydekker (1889), *Ischyrodon* was considered a synonym of *Pliosaurus* in several subsequent studies (De Stefani, 1903; von Huene, 1934; Wild, 1968). In turn, Tarlo (1960) was the first to discuss *I. meriani* as possibly being conspecific with *Liopleurodon ferox*. Such option was further considered or otherwise mentioned in Persson (1963), Geister (1998), Noè (2001), Barrientos-Lara, Fernández & Alvarado-Ortega (2015), and Madzia (2016).

Following the recent progress in the understanding of pliosaurid dental anatomy (*e.g.*, Madzia, 2016; Foffa et al., 2018; Zverkov et al., 2018; Madzia, Sachs & Lindgren, 2019), we redescribe the type of *I. meriani*, illustrate it in detail, and compare with teeth of other thalassophonean pliosaurids. We further study the specimen through multivariate analyses aimed to explore the morphospace occupation of *I. meriani* among thalassophoneans and to assess previously mentioned similarities between the type of *Ischyrodon* and *Pliosaurus* (Lydekker, 1889) and *Liopleurodon* (Tarlo, 1960). Finally, we discuss potential implications of *I. meriani* being conspecific with *L. ferox*, and comment on the taxonomic status of *Liopleurodon*.

## Geological Setting

NMB L.D.37, the type of *Ischyrodon meriani*, has a series of labels mentioning the ‘Eisenrogenstein’ from ‘Wölflinswyl’. In the following, we shortly discuss the geology and the geographic origin and then the stratigraphic position.

### Regional geology and the geographic origin

**Figure 1:**
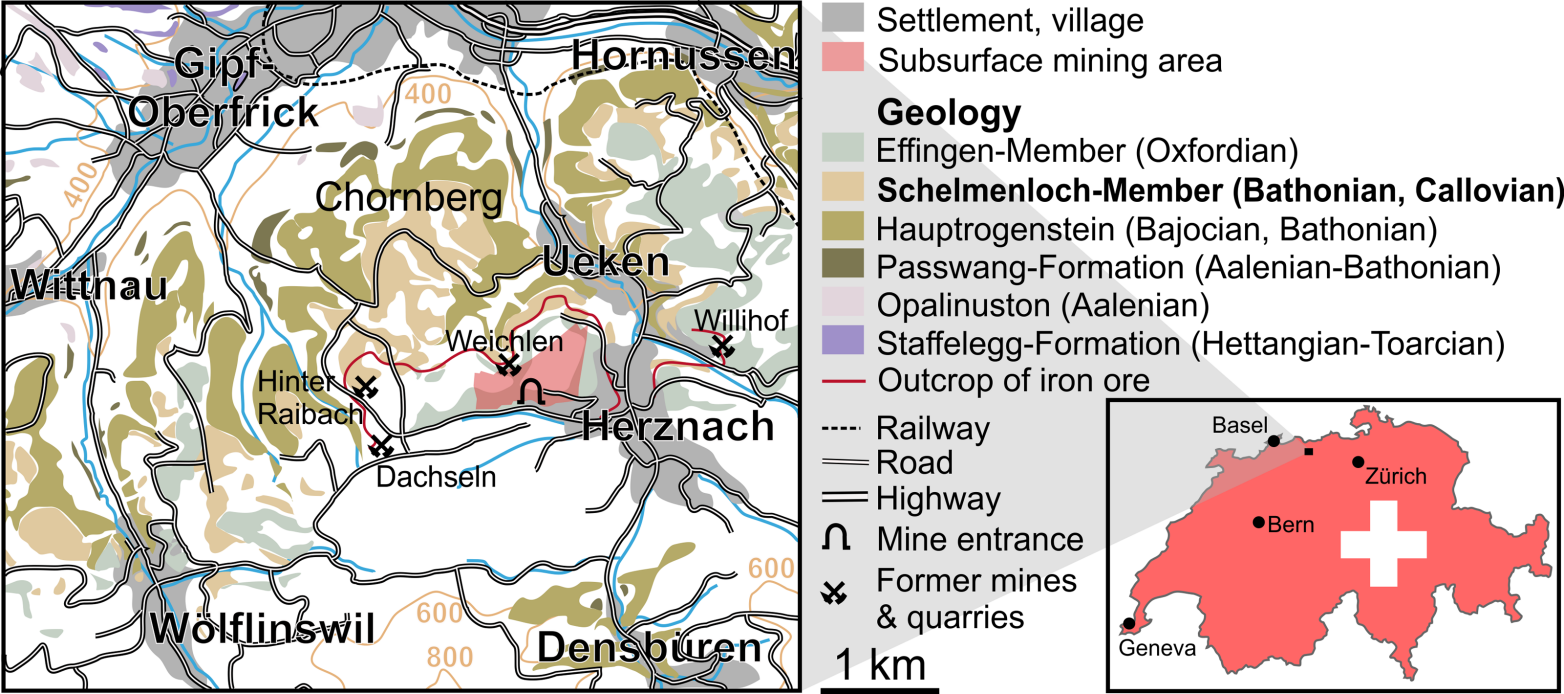
Geological map of the Frick region depicting the position of the village of Wölflinswil as well as that of the former iron mine at Herznach. The map was redrawn using various maps from SwissTopo (https://map.geo.admin.ch) and from Hüsser (1996).

‘Wölflinswyl’, now spelled Wölflinswil, is located in the Swiss canton Aargau near the villages of Frick and Herznach (Fig. 1) and, on a broader scale, between the towns of Basel and Zürich. In this area, Triassic and Jurassic sediments crop out. Here, we focus on the Jurassic since predominantly Jurassic sediments are exposed in this area. In this part of Switzerland, the Jura mountains, the Mesozoic strata are strongly folded and faulted tectonically in the south (‘Faltenjura’—folded Jura), whereas the area of interest lies in the ‘Tafeljura’ (table Jura), which was much less affected by the Alpidic orogeny (Herzog, 1956).

In the region between Frick (Gipf-Oberfrick), Hornussen, Wölflinswil and Herznach, Hettangian to Oxfordian sedimentary rocks reach the surface. While the strata of the Lower Jurassic Staffelegg-Formation (Reisdorf et al., 2011) are predominantly clayey with some marls and a few limestone beds, the Middle Jurassic sequence bears a great variety of marine sedimentary rocks including marls, oolites, crinoidal limestones, etc. (*e.g.*, Gonzalez & Wetzel, 1996).

Both the tooth and a rock sample contained in the same box show that the matrix is extremely rich in iron. This suggests that the tooth comes from the iron ore that was mined from around 1,200 until 1967 in various quarries and underground mines (Hüsser, 1996; Hüsser, 2002). Some of these former mines are indicated in the map (Fig. 1). Since the labels accompanying the specimen provide Wöflinswil as the locality, we must assume a locality that is closer to that village than to the neighboring village of Herznach, where parts of the biggest and most recent mine is still accessible. The former mines and quarries of Hinter-Raibach and Dachseln lie closest to Wöflinswil. According to Hüsser (1996), mines on the Chornberg—a small mountain between Herznach and Wöflinswil—were documented already in 1772. This fits reasonably well with the possible times of mining around 1800 at Hinter-Raibach or Dachseln. Nevertheless, we cannot rule out that the tooth was found outside of the mines in the scree.

### Stratigraphic position

Detailed accounts of the stratigraphic position of the iron ores in the Frick region have been published repeatedly (Jeannet, 1951; Jeannet, 1954; Fehlmann & Rickenbach, 1962; Gygi, 1981; Stössel, 2002; Stössel, Pika-Biolzi & Ungricht, 2010; Bitterli-Dreher, 2021). The original labels indicate ‘Rogeneisenstein’. Together with the sedimentary facies of the rock still attached to the fossil, the host rock is an iron oolite. In that region, these red iron oolites are known from the Callovian exclusively (Jeannet, 1951; Stössel, Pika-Biolzi & Ungricht, 2010; Bitterli-Dreher, 2021). The miners referred to the ‘Untere Erzbank’ (lower ore bed) and the ‘Oberes Erzlager’ (upper ore bed). These overlie the Hauptrogenstein, which is Bajocian to Bathonian in age. The Bajocian and Bathonian of Switzerland occasionally yields remains of marine reptiles such as plesiosaurs (Sachs, Klug & Kear, 2019) and large ichthyosaurs (CK, ongoing research), but usually, these sediments are grey and rich in echinoderms.

**Figure 2:**
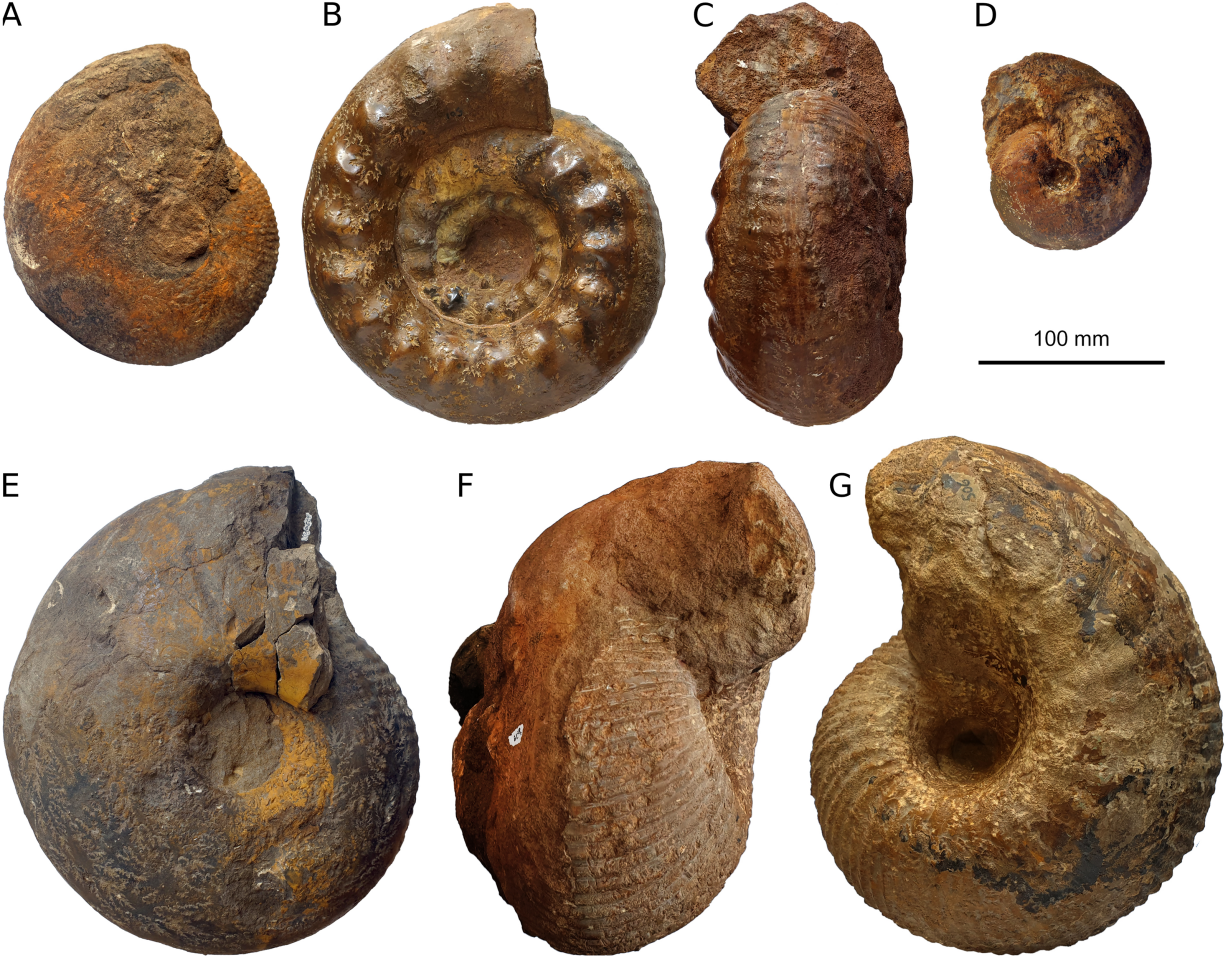
Ammonites from the iron ore mine at Herznach. All ammonites are depicted at the same scale. (A) *Macrocephalites jacquoti*, PIMUZ 16397, late Callovian, gracilis Zone. (B, C) lateral and ventral views of *Erymnoceras* cf. *coronatum*, PIMUZ 8079, Callovian. (D) *Cardioceras* sp., PIMUZ 8110, late Callovian. (E) *Macrocephalites tumidus*, early Callovian, enodatum Zone. (F, G) *Kamptokephalites* sp., PIMUZ 8078, Callovian.

Around Herznach and Wölflinswil, the two ore layers contain abundant and well preserved internal moulds of a diverse ammonite fauna (Jeannet, 1954; Gygi & Marchand, 1982). In particular, large specimens of *Erymnoceras*, *Kamptocephalites*, *Macrocephalites*, and phylloceratids have been found (Jeannet, 1951; Bitterli-Dreher, 2021), indicating a Callovian age (Fig. 2). The biostratigraphy of the Ifenthal Formation was portrayed by Bitterli-Dreher (2012), but the last comprehensive revision dates back to Jeannet (1951). In his dissertation, Hostettler (2014) revised the Middle Jurassic ammonites. Younger sedimentary rocks of Oxfordian age may also contain ooids and may be fairly rich in iron, but they tend to have a more greyish to greenish color.

## Methods

### Multivariate analyses

In order to explore the morphospace occupation of NMB L.D.37 among thalassophonean pliosaurids, and assess previously mentioned similarities of the type of *Ischyrodon* to *Pliosaurus* (Lydekker, 1889) and *Liopleurodon* (Tarlo, 1960), we performed cluster and principal coordinates analyses. We used the dataset of Bastiaans et al. (2021), which is a recent version of the one first published by Zverkov et al. (2018), and added NMB L.D.37. No further modifications have been provided. See Supplemental Information I for the matrix.

We used the same protocol as Zverkov et al. (2018) and Bastiaans et al. (2021). The analyses were carried out in the R statistical environment (RStudio Version 1.2.5033 R Studio Team, 2019). We applied a 50% completeness threshold to remove the influence of taxa which are based upon insufficiently complete or preserved specimens, scaled the data to equal variance and a mean of zero through subtraction of the mean value for each character and divided it by the standard deviation. Using the package cluster v2.1.0 we applied the Gower metric (Gower, 1971) to create a distance matrix. A cluster dendrogram was produced from the resulting matrix with the stats package, using the Ward.D2 method.

Then, we took the same matrix and used ape v5.3 (Paradis, Claude & Strimmer, 2004) to explore the dental morphospace occupation of *I. meriani* among the thalassophonean pliosaurids through a principal coordinates analysis. We applied the Gower metric and the Cailliez correction for negative eigenvalues. See Supplemental Information II and Supplemental Information III for the R code and extended results, respectively.

### Terminology of tooth orientation and morphology

We follow the terminology of tooth orientation of Smith & Dodson (2003): apical, toward the apices of the tooth crown or the tooth root; basal, toward the *cervix dentis*; distal, away from the tip of the snout; labial, toward the lips; lingual, toward the tongue; mesial, toward the tip of the snout.

The description of the characters of the outer enamel surface is provided using the nomenclature advocated by Zverkov et al. (2018) and followed in Páramo-Fonseca, Benavides-Cabra & Gutiérrez (2018), Madzia, Sachs & Lindgren (2019), Lukeneder & Zverkov (2020), Bastiaans et al. (2021), Madzia, Szczygielski & Wolniewicz (2021): apicobasal ridges, longitudinally running enamel ridges of variable apicobasal extent that can be developed around the entire crown circumference and are usually semicircular or triangular in cross-section; ridglets, subtle apicobasally-oriented enamel structures often developed between adjacent apicobasal ridges or on an unridged enamel surface; the appearance of ridglets varies from being very smooth to producing a distinct vermicular pattern (see Madzia, 2016: Fig. 7).

## Results

### Systematic paleontology

**Table:** 

Plesiosauria De Blainville, 1835
Pliosauridae Seeley, 1874
Thalassophonea Benson & Druckenmiller, 2014
*Ischyrodon meriani*von Meyer, 1838 (*nomen dubium*)
1838 *Ischyrodon* – von Meyer, p. 414
1838 *I. Meriani* – von Meyer, p. 414
1841 *Ischyrodon Meriani –* von Meyer, p. 183f
1845 *Ischyrodon* – Hoffmann, p. 327
1845 *Ischyrodon Meriani* – Hoffmann, p. 327
1846 *Ischyrodon Meriani* – Geinitz, p. 89
1847 *Ischyrodon* Merian – Giebel, p. 126
1847 *I. Meriani* Meyer – Giebel, p. 126, pl. p. 207
1853 *Ischyrodon*, Mérian – Pictet, p. 519
1853 *Ischyrodon Meriani* H. de Meyer – Pictet, p. 519
1856a *Ischyrodon Meriani –* von Meyer, p. 19ff
1856b *Ischyrodon Meriani –* von Meyer, p. 6, pl. 2, figs 1– 3
1883 *Ischyrodon Meriani –* Kiprijanow, p. 1ff
1885 *Ischyrodon Meriani* Myr – von Quenstedt, p. 212
1887–1890 *Ischyrodon Meriani* H. v. Meyer – Zittel, p. 497
1889 *Ischyrodon*, Meyer – Lydekker, p. 120ff
1903 *Ischyrodon* H. v. Meyer – de Stefani, p. 68f
1934 *Ischyrodon meriani* H. v. Meyer – von Huene, p. 45
1960 *Ischyrodonmeriani* – Tarlo, p. 165
1963 *Ischyrodon meriani* – Persson, p. 30
1968 *Ischyrodonmeriani* H. v. Meyer – Wild, p. 582
1998 *Ischyrodon meriani* H. v. Meyer, 1856 – Geister, p. 118
2001 *Ischyrodon meriani* von Meyer – Noè, p. 59f
2015 *Ischyrodonmeriani* von Meyer, 1838 – Barrientos-Lara et al., p. 294
2016 *Ischyrodon meriani* – Madzia, p. 27

**Figure 3:**
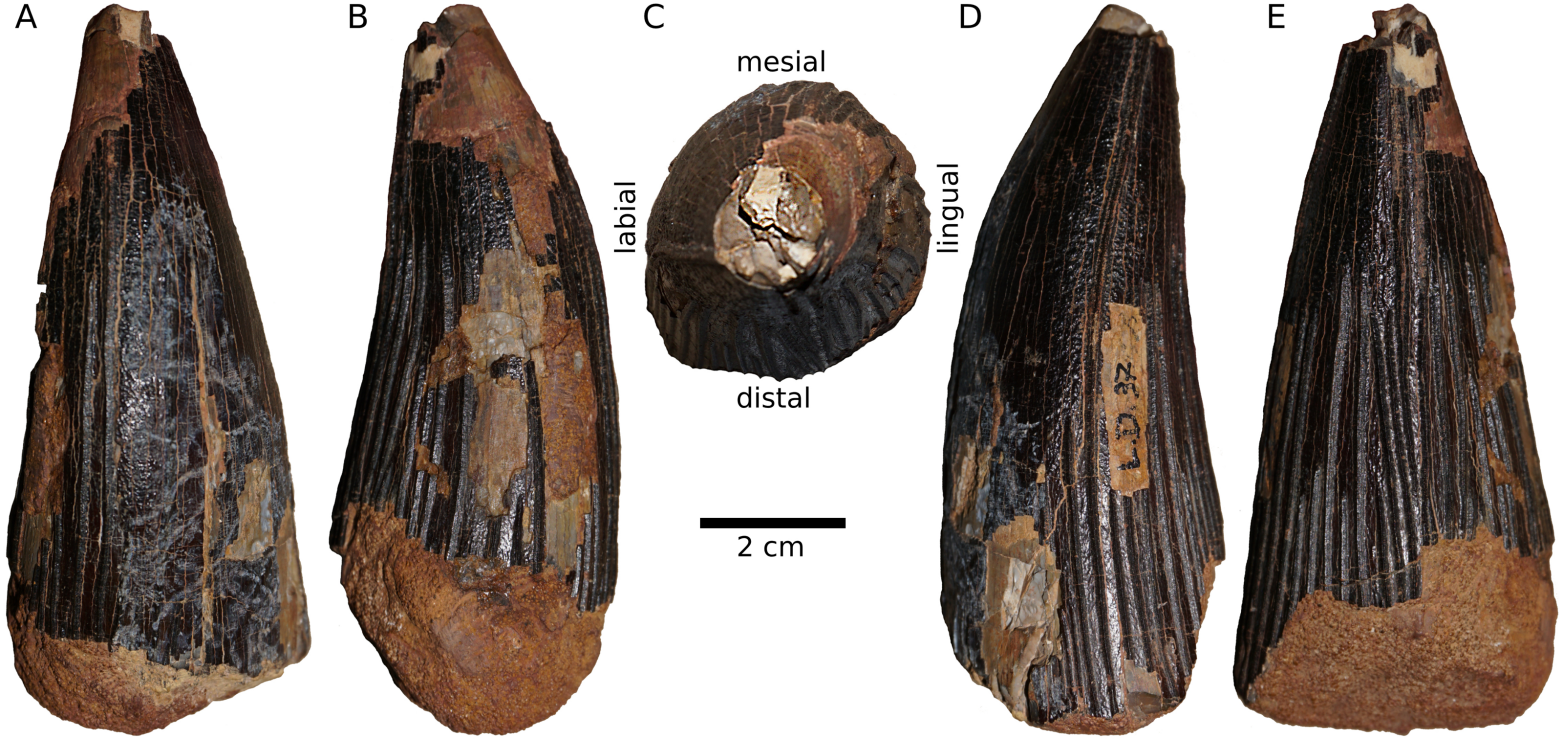
NMB L.D.37, the type of *Ischyrodon meriani*, in (A) mesial, (B) lingual, (C) apical, (D) labial, and (E) distal view.

**Type specimen.** NMB L.D.37, an isolated tooth crown (Fig. 3).

**Locality and age.** Wölflinswil, near the villages of Frick and Herznach, Canton of Aargau, Switzerland; most likely Callovian, Middle Jurassic.

**Description and comparisons.** The size, stoutness, and character of the linguodistal curvature of the tooth crown indicate that it originates from the anterior half of the right maxilla or the left dentary. The preserved part of the crown of NMB L.D.37 is approximately 107.8 mm high (total crown height is estimated to reach ∼110 mm) and its maximum cross-sectional diameter is ∼50 mm. The basal cross-section is sub-circular, similar to the condition observed in *Acostasaurus pavachoquensis* (Gómez-Pérez & Noè, 2017), *Brachauchenius lucasi* (Liggett et al., 2005; Albright, Gillette & Titus, 2007), *Monquirasaurus boyacensis* (Hampe, 1992; Noè & Gómez-Pérez, 2021), *Kronosaurus queenslandicus* (McHenry, 2009), *Liopleurodon ferox* (Noè, 2001), *Marmornectes candrewi* (Ketchum & Benson, 2011a), *Megacephalosaurus eulerti* (Madzia, Sachs & Lindgren, 2019), *Pachycostasaurus dawni* (Cruickshank, Martill & Noè, 1996; Noè, 2001), *Peloneustes philarchus* (Ketchum & Benson, 2011b), ‘*Pliosaurus*’ *andrewsi* (Tarlo, 1960; Noè, 2001; Zverkov et al., 2018), ‘*Polyptychodon*’ *hudsoni* (D. Madzia, personal observation, 2018), *Sachicasaurus vitae* (Páramo-Fonseca, Benavides-Cabra & Gutiérrez, 2018), *Simolestes vorax* (Tarlo, 1960; Noè, 2001; Zverkov et al., 2018), the assemblage historically assigned to ‘*Polyptychodon interruptus*’ (Madzia, 2016), and other isolated pliosaurid teeth with indeterminate taxonomic status and phylogenetic placement (*e.g.*, Kear et al., 2014; Zverkov, 2015; Madzia & Machalski, 2017; Zverkov et al., 2018; Lukeneder & Zverkov, 2020; Solonin, Vodorezov & Kear, 2021; Bastiaans et al., 2021). It differs from *Gallardosaurus itturraldei* (Gasparini, 2009), *Luskhan itilensis* (Fischer et al., 2017), *Makhaira rossica* (Fischer et al., 2015), *Pliosaurus* (*e.g.*, Andrews, 1913; Knutsen, 2012; Benson et al., 2013), *Stenorhynchosaurus munozi* (Páramo-Fonseca et al., 2016), and some other tooth crowns (*e.g.*, Zverkov, 2015) that possess alternatively sub-trihedral (*G. itturraldei*, *L. itilensis*, *P. kevani*, *St. munozi*) to trihedral (*Mak. rossica*, *Pliosaurus* spp.), and trihedral-to-trapezoidal (Zverkov, 2015) crown cross-sections. The specimen NMB L.D.37 lacks carinae, unlike numerous Late Jurassic and Early Cretaceous pliosaurids that show one (*G. itturraldei*, *L. itilensis*, *P. kevani*, *S. munozi*), two (most species of *Pliosaurus*), or three (*Mak. rossica*) carinae (see Gasparini, 2009; Benson et al., 2013; Fischer et al., 2015; Zverkov, 2015; Páramo-Fonseca et al., 2016; Fischer et al., 2017; Zverkov et al., 2018).

The enamel of NMB L.D.37 shows prominent apicobasal ridges that are approximately triangular in cross-section, similar to the condition observed, for example, in *A. pavachoquensis*, *B. lucasi*, *Mo*. *boyacensis*, *L. ferox*, *Mak. rossica*, *Mar. candrewi*, *Pa. dawni*, *Pe. philarchus*, *Si. vorax*, *St. munozi*, some species of *Pliosaurus*, and some tooth crowns from the assemblage historically assigned to ‘*Polyptychodon*’ (Madzia, 2016; Zverkov et al., 2018). No ridge has been observed to be branching, as in most pliosaurids, but unlike in *Pa. dawni*, the Western Interior brachauchenines (Madzia, Sachs & Lindgren, 2019), and the ‘Maryevka pliosaurid’ (Zverkov et al., 2018). Enamel surface shows clear ridglets, similar to those in *L. ferox*, some species of *Pliosaurus*, some isolated pliosaurid tooth crowns from the Jurassic/Cretaceous boundary interval of Russia (Zverkov et al., 2018), some tooth crowns from the assemblage historically assigned to ‘*Polyptychodon*’ (Madzia, 2016; Zverkov et al., 2018), and *Sa. vitae* and the ‘Venezuelan pliosaurid’ (Bastiaans et al., 2021).

Finally, NMB L.D.37 shows a pattern of apicobasal ridges that is characterized by presence of three mesiolabially positioned ridges running through the entire apicobasal height of the tooth crown. Such pattern has been previously described for some tooth crowns from the mid-Cretaceous ‘*Polyptychodon*’ assemblage of East and South East England (Madzia, 2016: Fig. 5).

### Results of multivariate analyses

**Figure 4:**
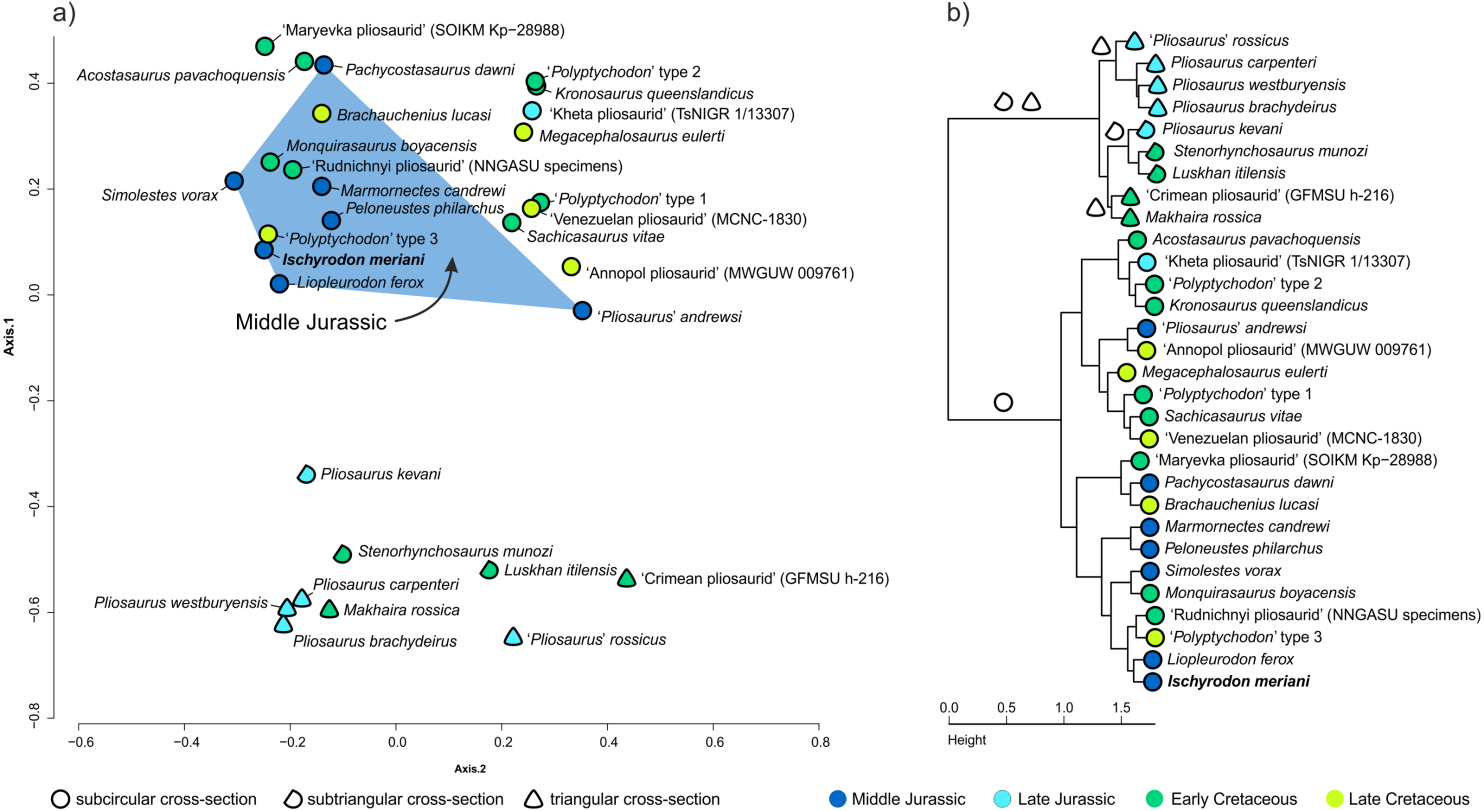
Multivariate analyses. Results of the principal coordinates analysis (A), which show the morphospace occupation of the type specimen of *Ischyrodon meriani* (NMB L.D.37) among Jurassic and Cretaceous pliosaurid taxa, through principal coordinates 1 and 2, and results of the cluster analysis (B). Graphic representation and color scheme of the results follow Zverkov et al. (2018).

The results of multivariate analyses are broadly congruent with those published by Zverkov et al. (2018) and Bastiaans et al. (2021) and we refer to these two studies for more detailed account of the results and interpretations thereof. The principal coordinates analysis (PCoA) placed NMB L.D.37 on the positive side of the first axis and the negative side of the second axis, in a close proximity to ‘*Polyptychodon*’ type 3 and *Liopleurodon ferox* (Fig. 4A). The cluster analysis placed NMB L.D.37 within the ‘conical’ morphogroup of the cluster dendrogram and grouped it with *Liopleurodon ferox* (Fig. 4B).

## Discussion

### When was the name *Ischyrodon meriani* formally established?

Owing to the fact that the publication, in which the name *I. meriani* was used for the first time, did not include detailed description of the type tooth, it has not been clear whether von Meyer’s (1838) contribution actually formally established the taxon name. For example, Tarlo (1960) stated that *I. meriani* remained a *nomen nudum* until von Meyer (1856a).

Article 12 of the *ICZN* (International Commission on Zoological Nomenclature, 1999) specifies that “[t]o be available, every new name published before 1931 must satisfy the provisions of Article 11 and must be accompanied by a description or a definition of the taxon that it denotes, or by an indication” (Art. 12.1.) and that a vernacular name, locality, geological horizon, host, label, or specimen do not in itself constitute a description, definition, or indication (Art. 12.3.).

In the original study, von Meyer (1838) noted the provenance of the tooth crown and mentioned that he would like to name it “*I. Meriani*” (also providing the name *Ischyrodon* in the same sentence). The description provided by von Meyer (1838) is limited to a statement that the type tooth surely belonged to a giant reptile (“Riesen-Saurus”) and that only *Mastodonsaurus* had similarly massive teeth; further noting that the teeth of the two taxa are very different (see von Meyer, 1838: 414, 415). Albeit brief and vague, this wording may qualify as “a description or a definition of the taxon that it denotes”. Owing to the absence of a universally followed set of rules that would govern the zoological nomenclature in the 1830s and the rather frequent use of the name *I. meriani* between 1838 and von Meyer’s (1856a) much more detailed description (von Meyer, 1841; Hoffmann, 1845; Geinitz, 1846; Giebel, 1847; Pictet, 1853), indicating that the authors mentioning the name treated it as an established taxon name, we are inclined to consider von Meyer’s (1838) contribution as the reference that established the name *I. meriani* as an available name in accordance with Article 12 of the *ICZN*. Such an interpretation renders *I. meriani* to be the historically earliest established taxon name of Pliosauridae.

### Is *Ischyrodon meriani* conspecific with *Liopleurodon ferox?*

The morphology and enamel character distribution of NMB L.D.37, as assessed through comparisons to teeth of other thalassophonean pliosaurids and by means of our multivariate analyses, show close similarities with *Liopleurodon ferox*. Such results reopen the question of the potential conspecificity of the specimens assigned to the two taxa. Tarlo (1960) was the first to note that the teeth of *I. meriani* and *L. ferox* are very similar but added that “[i]t is not certain from Meyer’s figure whether this assignment is altogether justified” (Tarlo, 1960: 166). Interestingly, Tarlo (1960) suggested that it was the name *I. meriani* that could be synonymized with *L. ferox*, rather than treating *L. ferox* as the potential junior synonym of *I. meriani*. Even though the material that is currently assigned to *L. ferox* is much better researched, much more complete, and taxonomically informative (see especially Andrews, 1913; Tarlo, 1960; Noè, 2001), it is *I. meriani* that would have nomenclatural priority (providing that *I. meriani* is declared diagnostic).

Still, despite that both these taxa originate from strata that are comparable in age, the fragmentary nature of the type tooth of *I. meriani*, combined with the observed similarities between the specimen and teeth of other thalassophoneans, even including some of the latest-diverging members of the clade (Madzia, 2016: Fig. 5), would make such taxonomic decisions unsubstantiated at present. We agree with Tarlo (1960), and some subsequent authors, such as Noè (2001: 27, 31), that there are good reasons to consider *I. meriani* and *L. ferox* to likely represent the same thalassophonean pliosaurid. However, the synonymization of the two names would have to be preceded with a morphometric and morphological assessment of the teeth of particular individuals assigned to *L. ferox* that would explore the dental variability within and between their jaws and reveal whether the tooth of *I. meriani* falls within that variability.

### Is *Ischyrodon meriani* a diagnostic taxon?

Despite its fragmentary nature, the type tooth of *Ischyrodon meriani* shows enough morphological characters to make a detailed comparison to teeth of other thalassophonean pliosaurid taxa feasible. At present, it appears impossible to list features, either autapomorphies or a unique combination of characters, that would enable the taxon to be diagnosed. Therefore, *I. meriani* is currently a *nomen dubium*, and the type tooth is best treated as Thalassophonea indet.

### Is *Liopleurodon ferox* a diagnostic taxon?

Sauvage (1873) established *Liopleurodon ferox* based on a single isolated tooth crown (BHN 3R 197) originating from the upper Callovian (uppermost Middle Jurassic) strata of Le Wast, France. The description of the morphology of the holotype has been generally considered adequate to treat *L. ferox* as a diagnostic pliosaurid taxon and numerous additional specimens have been assigned to it or associated with it since its establishment (see especially Noè (2001) for detailed discussion of the history of *L. ferox*, specimens referred to the taxon, and their stratigraphic range and geographic distribution). Some of these specimens have originated from distant localities (*e.g.*, Zverkov, Shmakov & Arkhangelsky, 2017) or are a few million years younger than the type tooth (*e.g.*, Madzia, Březina & Calábková, 2018).

Owing to the rather broad geographic and stratigraphic distribution of the specimens assigned to or associated with *L. ferox*, it remains unclear whether these are in fact representatives of a single taxon (or clade), or whether the *Liopleurodon* dental morphology does not simply reflect a ‘stage’ in pliosaurid evolutionary history.

Additionally, it remains unclear whether the *Liopleurodon* lineage comprises one taxon, *L. ferox*, or two taxa, *L. ferox* and *L. pachydeirus*. Tarlo (1960) was convinced that *L. pachydeirus*, established as *Pliosaurus pachydeirus* by Seeley (1869), represents a diagnostic taxon that can be distinguished from *L. ferox* based on the morphology (coarseness) of the apicobasal ridges of the tooth crowns, and the proportions of cervical vertebrae and femora, citing two specimens, the type CAMSM J.46912 and NHMUK PV R 2446, as representatives of the taxon. In the most recent revision of *Liopleurodon* by Noè (2001), however, *L. ferox* and *L. pachydeirus* are considered synonyms. According to Noè (2001: 172), “[t]he cervical vertebral characteristics utilised to distinguish *Liopleurodon pachydeirus* from *Liopleurodon ferox* (Tarlo, 1960) are non-diagnostic (based on Brown, 1981), and those of the teeth are here considered to represent individual variation”, though Noè (2001: 172) also notes that “[t]he holotype material of the two species cannot be directly compared, as that of *Liopleurodon ferox* is a single tooth, and that of *Liopleurodon pachydeirus* is a string of 17 cervical vertebrae including an atlas-axis complex”. Unfortunately, Noè’s (2001) PhD dissertation, though commonly referenced in the literature, remains formally unpublished and no detailed revision of *Liopleurodon* has been provided since the early 2000s.

Even if the differences observed between the specimens assigned to *L. ferox* and those considered to form *L. pachydeirus* represent individual variation, which indeed appears plausible, *Liopleurodon* should be still subjected to detailed reevaluation. As per Noè (2001: 142), the crown ornamentation of *Liopleurodon* is variable. It may therefore be needed that an *ICZN* petition is filed to replace BHN 3R 197, the type of *L. ferox*, with a different specimen, for example NHMUK PV R 3536 that is reasonably complete and has been commonly treated in comparative studies as a ‘typical’ representative of *L. ferox*.

## Conclusions

*Ischyrodon meriani* is an obscure pliosaurid taxon based on a single tooth crown (NMB L.D.37) unearthed from the Callovian (Middle Jurassic) of Wölflinswil, Canton of Aargau, Switzerland. Despite its nearly two-century-long research history, the type of *I. meriani* is surprisingly poorly known. Previous studies have associated the material with *Pliosaurus macromerus* and *Liopleurodon ferox*, though neither of the two hypotheses have been thoroughly explored.

We have redescribed the type tooth of *I. meriani*, compared its morphology to that of other thalassophonean pliosaurids, and assessed its outer enamel structural elements through multivariate analyses. Our study supports close similarities of *I. meriani* with *Liopleurodon ferox* though comparable structures have also been observed in some late-diverging brachauchenines. While we are inclined to consider *I. meriani* conspecific with *L. ferox*, the synonymization of both these names, which would make *I. meriani* the proper name for the taxon, would be unsubstantiated at present. Both, *I. meriani* and *L. ferox* are based on single tooth crowns whose morphologies do not appear to be unique enough to enable the taxa to be adequately diagnosed. We refrain from making far-reaching decisions regarding *L. ferox*; however, *I. meriani* is treated here as a *nomen dubium*.

Following the redescription of *I. meriani*, we comment on the taxonomic status of *Liopleurodon ferox*. We posit that a detailed reevaluation of *Liopleurodon* appears to be needed and replacement of its type specimen may be necessary to maintain the current understanding of the taxon.

## Supplemental Information

10.7717/peerj.13244/supp-1 Supplemental Information 1 Character matrix for multivariate analyses

10.7717/peerj.13244/supp-2 Supplemental Information 2 R code for multivariate analyses

10.7717/peerj.13244/supp-3 Supplemental Information 3 Extended results of the multivariate analyses

## Institutional abbreviations

BHN: Musée Boulogne-sur-Mer, France
CAMSM: Sedgwick Museum of Earth Sciences, University of Cambridge, Cambridge, UK
GFMSU: Geological Faculty of Lomonosov Moscow State University, Museum at the academic base named after Prof. A. A. Bogdanov, Bakhchisaray district, Crimea
MCNC: Museo de Ciencias Naturales de Caracas, Caracas, Venezuela
MWGUW: Stanisław Józef Thugutt Geological Museum, Faculty of Geology, University of Warsaw, Warsaw, Poland
NHMUK: Natural History Museum, London, UK
NMB: Naturhistorisches Museum Basel, Basel, Switzerland
NNGASU: Museum of Nizhny Novgorod State University of Architecture, Building and Civil Engineering, Nizhny Novgorod, Russia
PIMUZ: Paläontologisches Institut und Museum, Zürich, Switzerland
SOIKM: Samara Regional History and Local Lore Museum named after P.V. Alabin, Samara, Russia
TsNIGR: Central scientific research geological survey museum named after Academician F.N. Chernyshev, St Petersburg, Russia

## Additional Information and Declarations

### Data Availability

The following information was supplied regarding data availability: The raw data are available in the Supplementary Files. The raw data have been obtained from a previous interation of the dataset (Bastiaans et al., 2021), first published by Zverkov et al. (2018). The only addition in the present study is the specimen NMB L.D.37 which is housed at the collections of the Naturhistorisches Museum Basel, Basel, Switzerland. For data on other specimens, see Zverkov et al. (2018) and Bastiaans et al. (2021).
