# Supplementary material for: Historical significance and taxonomic status of Ischyrodon meriani (Pliosauridae) from the Middle Jurassic of Switzerland
Source: PeerJ. 2022 Apr 7;10:e13244. doi: 10.7717/peerj.13244 (PMC8995022; doi:10.7717/peerj.13244)
Supplement: Supplemental Information 1 [file peerj-10-13244-s001.docx]

**Supplementary Information I**

**Character matrix**

| TAXON | C1 | C2 | C3 | C4 | C5 | C6 | C7 | C8 | C9 | C10 | C11 |
| --- | --- | --- | --- | --- | --- | --- | --- | --- | --- | --- | --- |
| *Marmornectes candrewi* | 25.3 | 2.976470588 | 0 | 1 | 0 | 0 | 0 | 1 | 1 | 0 | 1 |
| *Pachycostasaurus dawni* | 33 | 1.736842105 | 0 | 1 | 1 | 0 | 0 | 0 | 1 | 0 | NA |
| ‘*Pliosaurus*’ *andrewsi* | 41.1 | 1.763948498 | 0 | 0 | 0 | 0 | 1 | 1 | 0 | 0 | 2 |
| *Peloneustes philarchus* | 31.3 | 2.576131687 | 0 | 1 | 0 | 0 | 0 | 1 | 0 | 0 | 1 |
| *Simolestes vorax* | 82.4 | 2.559006211 | 0 | 1 | 0 | 0 | NA | 0 | 1 | 2 | 1 |
| *Liopleurodon ferox* | 81 | 2.436090226 | 0 | 1 | 0 | 0 | NA | 1 | 0 | 1 | 2 |
| *Anguanax zignoi* | 18 | 1.5 | NA | NA | NA | 0 | 0 | 2 | NA | NA | NA |
| *Gallardosaurus itturraldei* | NA | NA | 1 | 0 | NA | 1 | 0 | NA | NA | NA | NA |
| *Pliosaurus brachydeirus* | NA | 2.2 | 2 | 1 | 0 | 2 | 1 | 2 | 0 | 1 | 0 |
| *Pliosaurus kevani* | 48 | 1.655172414 | 1 | 1 | 0 | 1 | 1 | NA | 0 | 1 | NA |
| *Pliosaurus westburyensis* | 110 | 2.2 | 2 | 1 | 0 | 2 | 1 | 2 | 0 | 1 | 0 |
| *Pliosaurus carpenteri* | 100 | 2 | 2 | 1 | 0 | 2 | 1 | 2 | 0 | 1 | 0 |
| ‘*Pliosaurus*’ *rossicus* | 130 | 3.25 | 2 | 0 | 0 | 2 | 1 | 2 | 0 | 1 | 0 |
| ‘Kheta pliosaurid’ | 63 | 1.96875 | 0 | 0 | 0 | 0 | 0 | 0 | 1 | 0 | 1 |
| ‘Maryevka pliosaurid’ | 28 | 1.866666667 | 0 | 1 | 1 | 0 | 0 | 0 | 2 | 1 | 1 |
| ‘Rudnichnyi pliosaurid’ | 75 | 1.88 | 0 | 1 | 0 | 0 | 0 | 0 | 0 | 1 | 2 |
| ‘Crimean pliosaurid’ | 22 | 1.466666667 | 2 | 0 | 0 | 2 | 1 | 2 | 0 | 0 | NA |
| *Makhaira rossica* | 48 | 3 | 3 | 1 | 0 | 2 | 1 | 2 | 0 | 0 | NA |
| *Luskhan itilensis* | NA | NA | 1 | NA | 0 | 1 | 1 | 2 | 0 | 0 | NA |
| *Acostasaurus pavachoquensis* | NA | NA | 0 | 1 | 0 | 0 | 0 | 0 | 2 | 0 | NA |
| *Stenorhynchosaurus munozi* | NA | NA | 1 | 1 | 0 | 1 | 1 | 2 | 0 | 0 | 0 |
| *Kronosaurus queenslandicus* | 100 | 2 | 0 | 0 | 0 | 0 | 0 | 0 | 2 | 0 | NA |
| *Monquirasaurus boyacensis* | 90 | 2.5 | 0 | 1 | 0 | 0 | 0 | 0 | 0 | NA | 1 |
| *Megacephalosaurus eulerti* | 89 | 1.854166667 | 0 | 0 | 1 | 0 | 0 | 0 | 0 | 0 | 0 |
| *Brachauchenius lucasi* | 25 | 2 | 0 | 1 | 1 | 0 | 0 | 0 | 0 | 0 | NA |
| ‘*Polyptychodon*’ type 1 | 70 | 2 | 0 | 0 | 0 | 0 | 0 | 1 | 0 | 0 | 1 |
| ‘*Polyptychodon*’ type 2 | 70 | 2 | 0 | 0 | 0 | 0 | 0 | 0 | 2 | 0 | 1 |
| ‘*Polyptychodon*’ type 3 | 95 | 1.9 | 0 | 1 | 0 | 0 | 0 | NA | 0 | 1 | NA |
| ‘Annopol pliosaurid’ | 36 | 1.8 | 0 | 0 | 0 | 0 | 1 | 0 | 0 | 0 | 1 |
| *Sachicasaurus vitae* | 90 | 1.285714286 | 0 | 0 | 0 | 0 | 0 | 1 | 0 | 1 | NA |
| ‘Venezuelan pliosaurid’ | 30 | 1.5 | 0 | 0 | 0 | 0 | 0 | NA | 0 | 1 | NA |
| *Ischyrodon meriani* | 110 | 2.2 | 0 | 1 | 0 | 0 | 0 | 1 | 0 | 1 | NA |
